# siRNA-guided DNA-methylation at a sucrose transporter gene potentially contributes to climatic plant adaptation

**DOI:** 10.1371/journal.pbio.3003919

**Published:** 2026-07-30

**Authors:** Jianqiu Li, Min Song, Qiyu Xu, Yunjia Tang, Johannes Liesche

**Affiliations:** 1 College of Life Sciences, Northwest A&F University, Yangling, China; 2 State Key Laboratory of Crop Stress Biology for Arid Areas, Northwest A&F University, Yangling, China; 3 College of Landscape Architecture and Forestry, Qingdao Agricultural University, Qingdao, China; 4 Institute of Biology, University of Graz, Graz, Austria; Max Planck Institute for Biology Tübingen, GERMANY

## Abstract

Climatic adaptation of plants involves the adjustment of carbon transport to achieve an optimal balance between carbon assimilation and carbon utilization. This study describes a potential molecular mechanism of this adjustment by investigating the climate-dependent regulation of *SUCROSE TRANSPORTER 2* (*SUC2*) in the model plant *Arabidopsis thaliana*. SUC2 is responsible for loading photosynthetically-produced sucrose into the leaf vascular system. Its protein and mRNA abundance in *A. thaliana* natural accessions were found to correlate with leaf sucrose export and summer temperature. Exploration of methylome data and epigenome editing experiments suggested that epigenetic marks on *SUC2’s* exon 2 contributed to the climate-dependent expression of *SUC2*. Two siRNAs were identified as mediators of *SUC2* methylations and their respective origins localized to the *SUC5* and *SUC8* genes. The analysis of plants with reduced or enhanced *SUC5* or *SUC8* transcriptional activity corroborated the link to *SUC2* methylation. Besides illustrating a novel regulatory strategy of carbon export from leaves, our results establish exon methylation mediated by gene-derived siRNAs as yet another potential mechanism for plant adaptation.

## Abstract 2

Plant adaptation to climate requires precise regulation of carbon export to balance assimilation and utilization. This study shows siRNA-guided exon methylation of a sucrose transporter gene that modulates its expression with temperature, revealing a mechanism linking epigenetics to adaptive carbon transport.

## Introduction

In plants, a fine regulation of carbon transport is essential to achieve an optimal balance of carbon assimilation in source tissues and carbon utilization in sink tissues [1]. This balance is especially important for adaptation to local climatic conditions [2]. However, the complexity and highly conserved genetic architecture of carbon transport-related traits have prevented gaining a clear picture of the molecular mechanism underlying the adaptation of carbon transport to a plant’s environmental conditions [3–5]. Understanding this mechanism could facilitate the breeding of climate-resistant crops [6]. Our investigation focuses on a key step in whole-plant carbon transport, the export of sucrose from leaves. Sucrose is the product of photosynthesis that serves as the stable transport unit of carbon and energy in plants [1]. The rate of sucrose export from leaves defines the carbon flow to sink organs and influences photosynthesis rates via feedback regulation [7]. In the model plant *Arabidopsis thaliana* and most crops, the sucrose export rate from a given leaf is mainly determined by the activity of the sucrose transporter that loads sucrose into the transport cell complexes of the phloem vascular tissue in leaf veins [8–10]. This sucrose transporter is called SUC2 in *A. thaliana* and SUT1 in most other plants [9].

Previous studies have investigated adaptive strategies regarding the growth rate and photosynthetic capacity of *A. thaliana* natural accessions [11–13], whose broad geographic distribution and associated bioinformatic resources make them a powerful tool for investigating mechanisms of adaptation and acclimation [14,15]. Generally, accessions from hot climates, as indicated, e.g., by summer temperatures, were found to have low photosynthesis rates, absolute growth rates and rosette dry mass while the opposite was true for accessions growing in colder climates [11,13,16]. Accordingly, we hypothesized that the sucrose export rate is negatively correlated with climate factors such as summer temperature.

Natural accessions display their inherent growth strategies to a large degree, even when grown under control conditions [17–19], revealing a genetic or epigenetic basis. Genetic mechanisms of climate-dependent gene regulation include, for example, variation in transcription factor binding sites [20]. Of the different types of epigenetic regulation, RNA-directed DNA methylation (RdDM) and gene body methylations appear to be the most relevant in the context of adaptation [21–24]. The RdDM pathway, in which a short interfering RNA (siRNA) guides the methylation machinery to its complementary target site [25], is recognized to mainly confer CHH methylation (C followed by A, T, or C) at short transposons in euchromatic regions, as well as the edges of long heterochromatic transposons [26,27]. Positioning of such transposons close to promoter sequences has been linked to prevention of transcription factor binding [23]. CHH methylations generally exert a negative influence on transcriptional activity [28]. A specific example of adaptation through RdDM is the climate-dependent methylation of transcription factor *ICE1* [29]. CHH methylations and *ICE1* transcript levels were found to correlate with winter temperatures of *A. thaliana* natural accessions [29]. CG methylations, which are typically not conferred via RdDM, are mostly found on coding regions [22,30]. These so-called gene body methylations appear to activate transcription [28] and total levels in *A. thaliana* accessions were found to scale with temperature and other environmental parameters [22,30]. No link to climatic conditions has been observed for the third type of cytosine methylations, the CH methylations [22,29].

In this study, we explore the climate association of leaf sucrose export rates and *SUC2* sequence, transcript levels, and methylation levels. Upon observing climate-associations of export rates and of *SUC2* transcript and methylation levels, we investigated the mechanism of how the *SUC2* methylations are established. In Arabidopsis, transgenerational inheritance via CHH methylations is facilitated by siRNAs during reproduction [31]. The results show how epigenetic regulation of *SUC2* contributes to the climate-adaptation of leaf sucrose export.

## Results

### Sucrose export and *SUC2* transcript abundance correlate with climate variables

To test if leaf sucrose export is associated with climatic factors, we selected natural accessions representing a broad range of summer temperatures in their habitats (S1 Table). Maximal summer temperature was previously found to have the strongest association with *A. thaliana* growth strategy, mainly indicated by the life cycle period, among common climatic variables [11]. We used “T_max_ summer” of the WorldClim 2 dataset as principal variable, which constitutes the hottest temperature of the warmest quarter averaged between 1970 and 2000 [32]. This summer temperature correlates with other climatic factors such as summer solar radiation, precipitation, and water vapor pressure (S2 Table).

**Fig 1:**
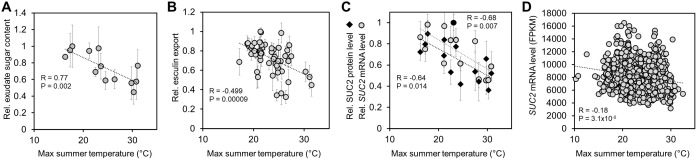
Climate-dependence of leaf sucrose export. Phloem exudate sucrose content **(A)**, export of the fluorescent sucrose-analog esculin **(B)**, *SUC2* mRNA and SUC2 protein level **(C)**, and *SUC2* mRNA level **(D)** plotted against the maximal temperature during summer of various natural accessions. Esculin export was calculated as 1 divided by the esculin fluorescence in the labeled source leaf after the loading period, meaning that higher values indicate a higher esculin export rate. Data on exudate sucrose content and esculin export for selected accessions are resolved in S1 Fig. mRNA levels were determined by qPCR **(C)** or transcriptomics analysis **(D)**. FPKM: fragments per kilobyte million. All samples were source leaves of 4-week-old plants grown under controlled long day-conditions at 21 °C **(A–C)** or 22 °C **(D)**. Bars indicate standard deviation. Lines indicate significant Pearson correlation (*P* < 0.05 in A–C, Bonferroni-corrected *P* < 7 × 10^−5^ in D). Number of biological replicates: 5 **(A)**, ≥7 **(B)**, 6 **(C)**. Number of accessions: 13 **(A)**, 56 **(B)**, 14 **(C)**, 656 **(D)**. Accessions are listed in S1 Table. Original geographic distribution of *A. thaliana* accessions used in figure 1 is shown in S1D Fig. The data underlying this figure can be found in the supplemental information file S1 Data.

Measurements of sucrose content in the leaf phloem exudate, one indicator of sucrose export activity [33], showed a negative correlation with summer temperatures (Fig 1A). A second indicator, leaf export of the fluorescent sucrose analogue esculin [34], confirmed this relationship (Fig 1B). Both measures of sucrose leaf export showed a significant correlation (ρ = −0.5729, *P* = 0.0000137; S1 Fig). Measurement of *SUC2* mRNA and protein levels in 14 accessions representing various climates (S1 Table) showed, like the sucrose export rate indicators, a negative correlation with summer temperature (Fig 1C). *SUC2* mRNA levels extracted from transcriptomics data of 656 accessions for which summer temperature data were available confirmed a significant correlation, although with a relatively small correlation coefficient (Fig 1D).

### Epigenetic modifications of *SUC2* correlate with summer temperature

**Fig 2:**
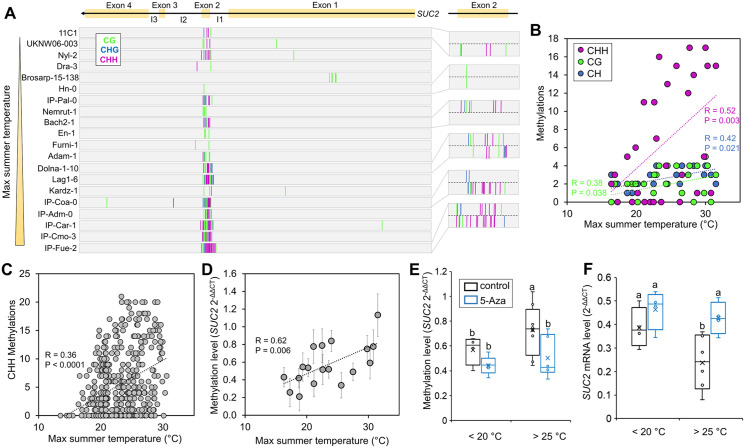
Association of epigenetic modifications with summer temperature. **A)** Visualization of cytosine methylations (CHH, CG and CH) on the *SUC2* gene in 20 accessions arranged according to their max summer temperatures. The right side shows the exon 2 at higher magnification and with methylation sites on the two strands resolved. I – intron. The corresponding screenshot from the 1,001 epigenome database is provided in S2A Fig. **B)** Number of cytosine methylations on *SUC2* exon 2 plotted against max summer temperatures in 30 accessions. **C)** *SUC2* exon 2 methylation levels in 419 accessions plotted against max summer temperatures. The Partial Mantel test and linear regression results are provided in S2B–S2C Fig to control the effect of population structure. **D)** *SUC2* methylation level of different accessions determined by chop-PCR plotted against their respective summer temperatures. **E), F)** *SUC2* methylation level **(E)** and *SUC2* mRNA level **(F)** in control and 5-Aza-treated plants belonging to accessions with low summer temperatures (<20 °C) or high summer temperatures (>25 °C). All samples constituted source leaves of 4-week-old plants grown under controlled long day-conditions at 22 °C **(A–C)** or 21 °C **(D–F)**. Regression lines in **B–D** indicate significant Pearson correlation (*P* < 0.05). In box plots, average values are indicated by cross, median values by line, quartiles by box, and variability outside quartiles by error bars. Different letters indicate significant differences according to *t* test (*P* < 0.05). Number of biological replica*t*es: 5 **(D, E)**, 4 **(F)**. Number of accessions: 30 **(B)**, 419 **(C)**, 18 **(D)**, 10 **(E, F)**. A list of accessions is provided in S1 Table. The data underlying this figure can be found in the supplemental information file S1 Data.

Leaf sucrose export rate and *SUC2* mRNA and protein levels could be regulated through climate-dependent genetic variation of *SUC2*. However, the *SUC2* gene, including 5 kb upstream of the transcription start, showed no associations of sequence variations across 1,135 natural accessions with any of 205 climatic or geographic variables. This was tested using the GenoCLIM algorithm [16]. Alternatively, the observed correlation of summer temperature and *SUC2* mRNA levels could stem from epigenetic regulation of gene expression. We evaluated epigenetic modifications in the form of cytosine methylation on the gene body and an upstream region of 1.5 kb in 30 accessions (S1 Table) using data of the 1,001 Arabidopsis Methylome project [23]. Visualization of epigenetic marks on *SUC2* revealed a fascinating pattern in which DNA methylation is mostly concentrated on the small exon 2 and increase in number with higher summer temperature (Figs 2A and S2A).

Most methylations on the *SUC2* gene belong to the CHH type (Fig 2B). In the 30 accessions, CHH methylations showed the strongest correlation with summer temperature (R = 0.52, *P* = 0.003), while CG and CH methylations showed lower levels of association (CH: R = 0.42, *P* = 0.021; CG: R = 0.38, *P* = 0.038). Correlation analysis using data from 419 accessions, for which methylomic and transcriptomic data were available, confirmed a strong association of CHH methylation and summer temperature (R = 0.36, *P* < 0.0001) (Fig 2C). The other marks, CG and CH, showed lower levels of association (CG: R = 0.168, *P* = 0.0006; CH: R = 0.114, *P* = 0.019) that did not meet the Bonferroni criterium of significance of *P* < 0.00012. In contrast to the number of CHH methylations, the number of potential CHH sites on *SUC2* exon 2 was relatively constant across accessions, with 1,037 out of 1,089 accessions featuring 24 potential CHH sites and 52 accessions featuring 22 sites.

We further tested if the relationship between *SUC2* methylations and summer temperature was influenced by the population structure among the 419 accessions. In a partial Mantel test [35], we evaluated the relative contribution of max summer temperature in explaining the number of methylations, separating the CHH, CG, and CH contexts. The genetic distance matrix was generated from the first five principal components of 50,000 randomly selected SNPs. The partial Mantel test revealed a significant correlation of summer temperature with CHH methylations (R = 0.056; *P* = 0.0048) (S2B Fig). The correlation of population structure with CHH methylations was not significant (R = 0.021, *P* = 0.213). The results of the partial Mantel test were further supported by linear regression analysis, which similarly showed maximum summer temperature as significant predictor of SUC2 CHH methylation (β = 0.243, *P* = 0.0085) (S2C Fig). In contrast, no significant correlations were detected for CH or CG methylations (Partial Mantel test: CH R = 0.008, *P* = 0.307, CG R = 0.011, *P* = 0.279; linear regression: CH β = 0.017, *P* = 0.166, CG β = 0.038, *P* = 0.329) (S2B, S2C Fig). Together, these results consistently demonstrate an association of CHH methylation with max summer temperature that is not explained by population structure.

The correlation of *SUC2* methylation level and the summer temperature was also detected by chop-PCR, which does not discriminate between types of methylations, for 18 accessions (Fig 2D). Methylations can be artificially removed by exposing seeds to 5-aza-2′-de-oxycytosine (5-Aza) [29]. We treated 10 accessions, representing the low and high end of summer temperatures (S1 Table) with 5-Aza and used Chop-PCR to evaluate the effect. Methylation significantly decreased only in accessions originating from high temperature habitats, but not in those adapted from low temperature habitats (Fig 2E). After 5-Aza treatment no significant difference in *SUC2* methylation level was detected between accessions adapted to high or low summer temperature (Fig 2E).

Moreover, 5-Aza treatment indicated a link between *SUC2* methylation and mRNA level. 5-Aza treatment had a strong positive effect on *SUC2* mRNA levels in accessions that are adapted to summer temperatures above 25 °C, while accessions adapted to max temperatures below 20 °C only showed a modest increase that was not statistically significant (Fig 2F). Results match previous tests on *A. thaliana* Col-0 (max summer temperature 30.8 °C) that found *SUC2* to be significantly upregulated after treatment with 5-Aza [36].

Our results indicate a climate-dependent DNA methylation on exon 2 of *SUC2*, which could cause the climate-related differences in *SUC2* mRNA levels and leaf sucrose export observed above. In the following, we explore this hypothesis further by investigating how methylation on *SUC2* is established.

### *SUC2* methylations correlate with the abundance of two siRNAs

**Fig 3:**
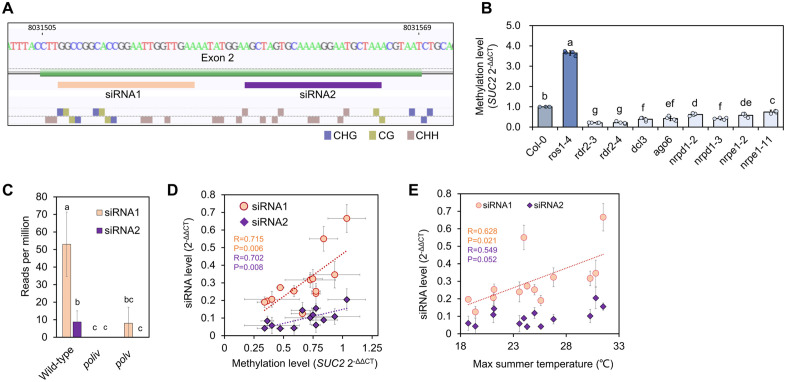
siRNA-guided methylation of SUC2. **A)** Illustration of exon 2 of *SUC2* with indicated positions of methylations and the regions complementary to two candidate siRNAs. **B)** *SUC2* methylation level in source leaves of the wild-type Col-0 accession and RdDM-related T-DNA-insertion mutants. ROS1 - REPRESSOR OF SILENCING1, RDR2 - RNA-DEPENDENT RNA POLYMERASE 2, DCL3 – DICERLIKE3, AGO6 - ARGONAUTE 6, NRPD1 - NUCLEAR RNA POLYMERASE D1, NRPE1 - NUCLEAR RNA POLYMERASE E1. See S3 Fig for mutant details. **C)** Abundance of the two candidate siRNAs as quantified by short RNA sequencing in flowers of wild-type Col-0 plants and mutants of RNA Polymerases IV (*nrpd1-4*) and V (*nrpe1-12*) in the Col-0 background. **D)** Candidate siRNA abundance in flowers determined by stem-loop PCR plotted against *SUC2* methylation level in leaves determined by chop-PCR in 13 accessions. **E)** Candidate siRNA abundance in flowers plotted against max summer temperature at the 13 accessions’ native habitats. Error bars indicate standard deviation. Different letters indicate significant differences according to *t* test (*P* < 0.05). Regression line indicates significant Pearson correlation (*P* < 0.05). Number of biological replicates: 4 **(A, C)**, 6 **(D, E)**. Accessions are listed in S1 Table. The data underlying this figure can be found in the supplemental information file S1 Data.

If indeed the methylations on exon 2 are responsible for the adaptation of *SUC2* activity, then a climate-sensitive mechanism for the establishment of methylations must exist. CHH methylations, the class of methylations dominating on the *SUC2* gene (Fig 3A), are established via the RdDM pathway that depends on target site-recognition with the help of 24nt-long siRNAs [25]. We confirmed that the RdDM pathway influences *SUC2* methylation with measurements on mutants of five genes with essential functions in RdDM and one RdDM-antagonist, the REPRESSOR OF SILENCING1 (ROS1) (S3 Fig). *SUC2* methylation levels were strongly increased in *ros1* mutants compared to the wild-type with the same background (Col-0) (Fig 3B). In contrast, *SUC2* methylation was reduced in mutant lines of the essential RdDM components RNA-DEPENDENT RNA POLYMERASE 2 (RDR2), DICERLIKE3 (DCL3), ARGONAUTE6 (AGO6), NUCLEAR RNA POLYMERASE D1 (NRPD1), NUCLEAR RNA POLYMERASE E1 (NRPE1) (Fig 3B), corroborating a link between RdDM and *SUC2* methylations. *SUC2* transcript levels were significantly increased in seven out of the eight RdDM pathway mutant lines featuring reduced *SUC2* methylation, while similar to wild-type in the other line (S3B, S3C Fig). Surprisingly, the *SUC2* transcript level was also increased in the strongly methylated *ros1* mutant (S3B Fig), illustrating that transcript levels can be influenced by processes beyond CHH methylation status. To test if CG or CH methylations could play a role, we checked *SUC2* transcript levels in mutants that cannot establish CH methylations (*cmt3, cmt2cmt3*) or CG methylations (*met1*), while maintaining an intact RdDM pathway [37,38]. *SUC2* transcripts levels similar to the Col-0 wild-type were detected in *cmt2* and *cmt2cmt3* mutants (S3D Fig). Only with additional disruption of the RdDM pathway in the *drm1drm2cmt2cmt3* mutant did *SUC2* transcript levels increase (S3D Fig). *met1* mutants lack all CG methylation, which was accompanied by fewer CHH reads in source leaves and less CHH methylation on exon 2 of *SUC2*, although there was not a robust effect on *SUC2* expression (*p* = 0.098) (S3D–S3F Fig).

Screening the sequence of *SUC2* exon 2 against a database of *A. thaliana* siRNAs yielded two 30 nt hotspots for binding of putative 24nt siRNAs that were identified in 33 libraries from different tissues of *A. thaliana* Col-0 or Ler-1 flowers (S4 Fig). Search for sequences complementary to the hotspots by nucleotide BLAST found one 24nt siRNA for each hotspot, termed siRNA1 and siRNA2 (Fig 3A). These siRNA’s involvement in RdDM was previously inferred from interaction with ARGONAUTE4 [39]. Expression of these siRNAs was found to depend on RNA Polymerase IV (Pol IV) and, to some degree, on RNA Polymerase V (Pol V) (Fig 3C). Most RdDM-related siRNAs in *A. thaliana* are transcribed by Pol IV [40]. Their processing is aided by the scaffold-RNA-producing Pol-V [41]. Quantification of siRNA levels by stem-loop PCR in flowers, the site of epigenetic imprinting, revealed a positive correlation of siRNA1 and siRNA2 abundance with *SUC2* methylation level, which we determined by chop-PCR, across accessions (Fig 3D). Moreover, siRNA1 abundance correlated with max summer temperatures (Fig 3E). siRNA2 showed a similar trend, with a relatively high Pearson correlation coefficient of 0.55 (*p*-value = 0.052) (Fig 3E). While chop-PCR does not discriminate between the type of methylation, this result confirms that siRNA-mediated methylation could act in the climatic adaptation of leaf sucrose export.

### Potential siRNA origins lie in sucrose transporter genes

**Fig 4:**
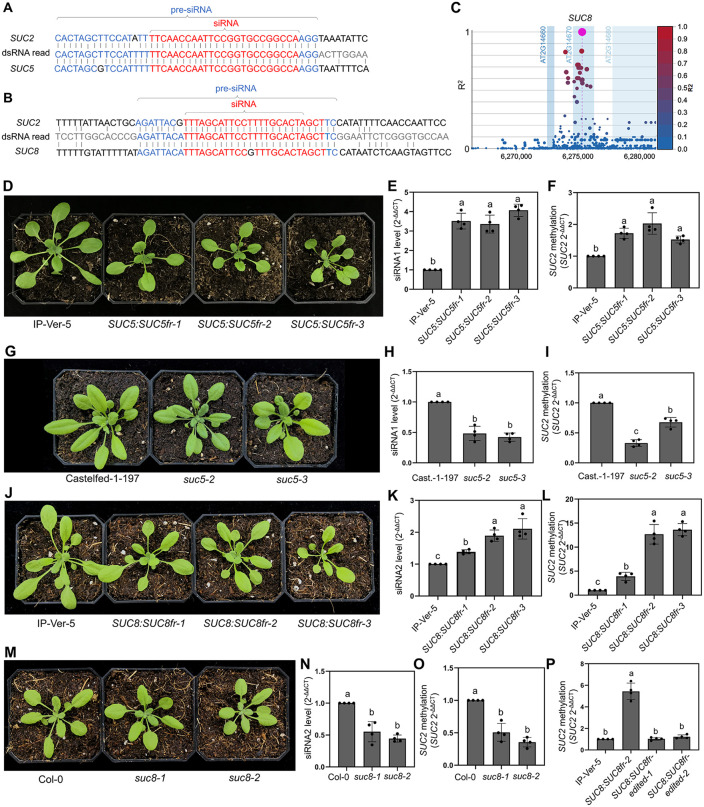
*SUC5* and *SUC8* as potential origins of siRNAs. **A)** Sequence alignment of pre-siRNA1 with *SUC2* and *SUC5*. **B)** Sequence alignment of pre-siRNA2 with *SUC2* and *SUC8*. Gray letters mark adapter sequences. **C)** Correlation of genetic variation with *SUC2* methylation as indicated by a genome-wide association study. The region around the *SUC8* gene is shown, which is one of the 9 loci that showed significant correlation (S3 Table). **D)** Photographs of IP-Ver-5 plants expressing an additional copy of the *SUC5* gene fragment including the siRNA1 sequence (*SUC5:SUC5fr*) in comparison to wild-type. **E)** siRNA1 levels in flowers of wild-type and *SUC5:SUC5fr* plants. **F)** *SUC2* methylation levels in source leaves of wild-type and *SUC5:SUC5fr* plants. **G)** Photographs of Castelfed-1-197 wild-type and *suc5* mutant lines. **H)** siRNA1 levels in wild-type and *suc5* plants. **I)** *SUC2* methylation levels in wild-type and *suc5* plants. **J)** Photographs of IP-Ver-5 wild-type plants and plants expressing a *SUC8* gene fragment containing the siRNA2 sequence (*SUC8:SUC8fr*). **K)** siRNA2 levels in flowers of wild-type and *SUC8:SUC8fr* plants. **L)** *SUC2* methylation levels in source leaves of wild-type and *SUC8:SUC8fr* plants. **M)** Photographs of Col-0 wild-type and *suc8* mutant lines. **N)** siRNA2 levels in flowers of wild-type and *suc8* plants. **O)** *SUC2* methylation levels in source leaves of wild-type and *suc8* plants. **P)** *SUC2* methylation levels in source leaves of wild-type, *SUC8:SUC8fr* and *SUC8:SUC8fr-edited* plants. All images show 4-weeks-old plants grown under long day-conditions. Error bars indicate standard deviation. Different letters indicate significant differences according to *t* test (*P* < 0.05). Number of biological replicates: 4. Information on the genetic background of plant lines is provided in S5 Fig. The data underlying this figure can be found in the supplemental information file S1 Data.

Analysis of double stranded RNA sequencing data from flowers of the *dcl234* mutant was used to identify sequences of the unprocessed pre-siRNAs corresponding to our candidate siRNAs. A whole-genome search using the putative 43nt pre-siRNA1 and the putative 34nt pre-siRNA2 indicated that they could originate from the *SUC2* gene itself (Fig 4). In addition, homology was observed between pri-siRNA1 and a section of the *SUC5* gene (Fig 4A) and between pri-siRNA2 and a section of the *SUC8* gene (Fig 4B). The potential siRNA origins lie in the exon 2 of *SUC5* and *SUC8*, respectively (S5 Fig).

The link between *SUC8* sequence and *SUC2* methylation was corroborated by a genome wide association study (GWAS) across 419 accessions using the number of methylations on exon 2 of *SUC2* as dependent variable. *SUC8* was one of the nine loci that showed significant correlation (Fig 4C; S3 Table). No association of SNPs within the *SUC5* gene was found.

We manipulated the abundance of siRNAs to verify their influence on *SUC2* methylation and validate their origin in the *SUC5*/*SUC8* genes. siRNA overexpressors were generated by introducing a gene construct containing the *SUC5* or *SUC8* promoter and the first part of the gene, ending just after the respective siRNA in an accession with relatively low siRNA levels (IP-Ver-5). A reduction of siRNA levels was achieved through knock-out/ knock-down of the *SUC5* or *SUC8* gene in accessions with high siRNA levels (Castelfed-1-197 for siRNA1 and Col-0 for siRNA2) (S5 Fig).

*SUC5:SUC5fr* plants exhibited reduced growth compared to wild-type plants (Fig 4D). siRNA1 levels were about three times higher than in the corresponding wild-type plants and *SUC2* methylation was significantly increased (Fig 4E–4F). In these *suc5* mutants, growth was not affected, but siRNA1 levels were reduced by about half compared to wild-type (Fig 4G, 4H). Moreover, the plants exhibited reduced levels of *SUC2* methylation (Fig 4I).

We obtained similar results for the plants in which *SUC8*/siRNA2 was targeted. The plant lines in which expressing an additional copy of a *SUC8* fragment including promoter and siRNA2 sequence with increased siRNA2 abundance showed increased *SUC2* methylation (Fig 4K, 4L). Their growth was reduced (Fig 4J). *SUC8* was knocked out (no detectable mRNA) in two lines with T-DNA insertions in the *SUC8* gene (S5 Fig) without affecting growth (Fig 4M). The consequence for siRNA2 abundance was a reduction by about 50% and a similar reduction in *SUC2* methylation levels (Fig 4N, 4O). In agreement with the effects of siRNA1 and siRNA2 overexpression on *SUC2* methylation, *SUC2* mRNA levels and the esculin loading rate were significantly reduced (S5C–S5F Fig). Correspondingly, SUC2 mRNA levels and esculin loading rate were increased in the mutant lines with reduced siRNA1 or siRNA2 levels (S5G–S5J Fig).

For siRNA2, we also succeeded in generating IP-Ver-5 lines that express an additional copy of the siRNA-containing part of the *SUC8* gene, just like for the overexpressor, but with base-edited siRNA2 sequence (S5K Fig). These lines did not show the increased *SUC2* methylation level observed in the overexpressors (Fig 4P). Instead, *SUC2* methylation, and *SUC2* transcript abundance, were similar to the level in wild-type plants (Figs 4P and S5L).

These results indicate a mechanistic link between *SUC5* activity and siRNA1 abundance as well as between *SUC8* activity and siRNA2 abundance. Moreover, they corroborate the link between abundance of the siRNAs and *SUC2* methylation. It should be noted that *SUC5* shares a high sequence homology with *SUC6*, and *SUC8* with *SUC7* and *SUC9*. In Col-0, all these *SUC*s show enrichment of on-exon CHH methylations similar to *SUC2* (S6 Fig).

### *SUC5* and *SUC8* activity could enact climate-dependent *SUC2* regulation

**Fig 5:**
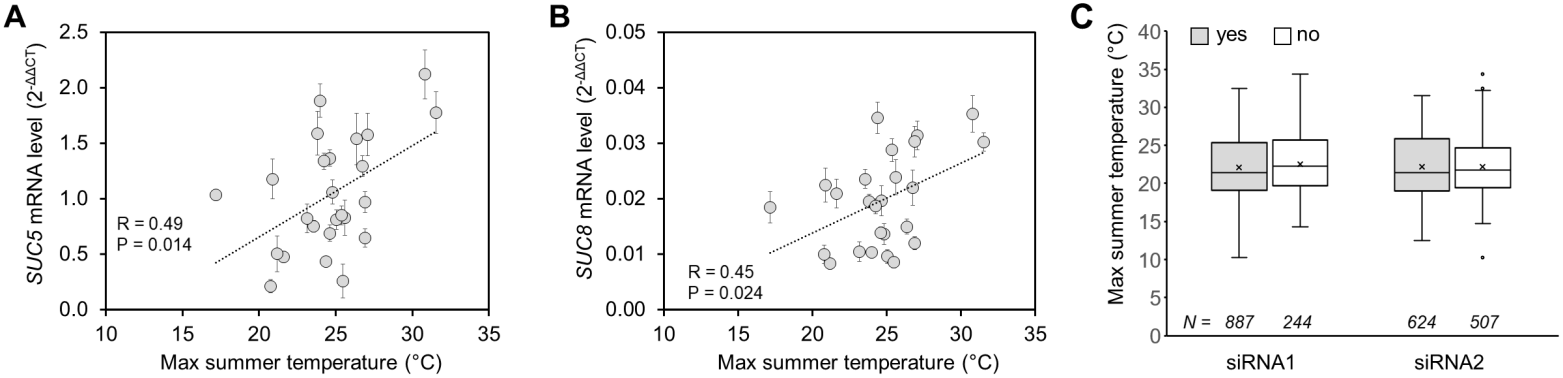
Climate-dependence of siRNA origin activity. **A, B)** mRNA levels of *SUC5* (A) and *SUC8* (B) in the flowers of 25 natural accessions plotted against max summer temperature at the accession’s habitat. Flower material was pooled from several plants. Error bars indicate standard deviation. Lines indicate significant Pearson correlation (*P* < 0.05). **C)** Max summer temperature in accessions that feature the exact siRNA sequences within *SUC5* (siRNA1) or *SUC8* (siRNA2) (yes), compared to max summer temperature in accessions with sequence differences (no). Average values are indicated by cross, median values by line, quartiles by box, and variability outside quartiles by error bars. Significance of differences was evaluated with *t t*est (*P* < 0.05). Number of biological replicates: 4 **(A, B)**. Accessions are listed in S1 Table. The data underlying this figure can be found in the supplemental information file S1 Data.

We measured *SUC8* and *SUC5* mRNA levels in flowers to evaluate if gene expression correlates with summer temperatures. Indeed, a strong correlation with summer temperatures was found for *SUC5* mRNA and for *SUC8* mRNA (Fig 5A, 5B).

In addition to gene activity variation, we investigated if the siRNA sequence variation could be associated with the climate. The siRNA origin sequences of the *SUC5* and *SUC8* genes showed some variation across accessions. The exact 24nt sequence that is complementary to the target region on *SUC2* was present in 887 out of 1,131 accessions in case of siRNA1, while the *SUC2*-complementary sequence of siRNA2 was present in 624 out of the 1,131 accessions. The max summer temperature of accessions containing the exact siRNA sequences was not significantly different for accessions with divergent sequences (*P* = 0.17 (siRNA1) and *P* = 0.93 (siRNA2); Bonferroni-limit of significance: *P* < 0.00004) (Fig 5C).

These results indicate that the gene activity of *SUC5* and *SUC8* could be responsible for climate-dependent siRNA production and *SUC2* methylation.

## Discussion

The rate of sucrose export from leaves directly influences photosynthetic activity and source-sink relations of plants [42]. The existence of a specific mechanism to adapt this parameter to environmental conditions is, therefore, not surprising. Our results indicate *SUC2* regulation to be an important mechanism for *A. thaliana* adaptation, just like it is for acclimation [8]. The strong correlation between *SUC2* mRNA levels and summer temperatures, as well as the climate-dependent *SUC2* methylations are clear indicators of *SUC2* being targeted to adjust the sucrose export rate from leaves in *A. thaliana* accessions. Our finding of leaf sucrose export being negatively correlated with summer temperature fits with the previously reported negative correlations of temperature and different photosynthesis- and growth-related phenotypic parameters, such as photosynthetic capacity, number of rosette leaves at bolting and absolute growth rate during vegetative development [13,16,17,43]. These links between temperature and carbon management relate to the shortening of the life cycle that allows Arabidopsis accessions to complete seed development before environmental conditions become adverse [11]. It should be noted that the maximal summer temperature, which most of our results relate to, serves as general climate indicator. It correlates with other relevant parameters, such as summer and spring precipitation or solar radiation (S2 Table) [11].

Carbon partitioning strategies exert a strong influence on ecosystem structure and function [44]. Accordingly, climate-adaptation of carbon partitioning has large implications for the global carbon budget [45,46]. Understanding the molecular mechanism underlying the adaptation of carbon partitioning can contribute to models that try to predict ecosystem performances under future climates. Moreover, the knowledge could facilitate the development of climate-resilient crops by offering pathways to the targeted manipulation of carbon transport [47,48]. Indeed, *Brassica napus* and tomato methylomes show methylations on exons of the sucrose transporter genes homologous to *SUC2* [49,50].

The epigenetic regulation of *SUC2* activity, with siRNA-mediated methylations of *SUC2*’s exon 2, exposes novel features of gene regulation in plant adaptation. However, it should be noted that this mechanism might not be the only way by which *SUC2* activity has adapted to the environmental conditions of the accessions. Genome-level adaptation could contribute to *SUC2* transcript level variation across accessions through climate-dependent polymorphisms on relevant transcription factor genes. Indeed, several of the transcription factors that potentially bind the *SUC2* promoter [51] show strong climate association [16]. Moreover, CHH methylations might also be influenced by CG methylations [37], which showed a presence on *SUC2*’s exon 2 that varied across accessions. The smaller effect on *SUC2* mRNA levels in plants with disrupted RdDM pathway compared to in plants where all methylations were removed by 5-Aza treatment could be due to a contributing role of CG methylations.

Epigenetic transcriptional regulation of CHH sites via RdDM has been previously implicated in climatic adaptation, but mainly as methylations on transposons [28,52] or promoters [23]. In addition, gene body methylations, which refer to CG methylations on the coding sequence, have been ascribed a role in adaptation, but these are established via a different mechanism and are associated with constitutive expression [28,53]. CHH methylations on the gene body have been predicted to act in climate-adaptation [30] and the climate-dependent *SUC2* methylations described here constitute a concrete example of this. RdDM-dependent regulation has been proposed to be more flexible than molecular adaptation through sequence polymorphisms [22], making it a preferred mechanism to increase the plant’s environmental resilience.

The temperature-correlated occurrence of CHH methylations on the *SUC2* gene led us to search for siRNAs that could mediate the establishment of these marks. The siRNAs identified here as facilitators of *SUC2* methylation appear to constitute an example for the recently discovered class of protein-coding gene-derived siRNAs [54], as the sequence homology of the precursor siRNAs indicates them to originate in the *SUC5* and *SUC8* genes, respectively. No evidentiary functions have been determined for *SUC5* and *SUC8* so far, as single gene knock-out mutants showed no obvious phenotype [55,56]. However, functional proteins were produced from both genes when expressed in *A. thaliana* mesophyll protoplasts [57]. Importantly, both are expressed in flowers [55], which include the tissues relevant for RdDM and the establishment of transgenerational inheritance [27,58]. While the details of siRNA biogenesis remain to be elucidated, it is clear that accessibility at the genome region covering a siRNA influences its expression [27]. Therefore, we measured siRNA abundance in plant lines, in which transcription of *SUC5* or *SUC8* was either repressed or hyperactivated. While *SUC5* and *SUC8* overexpression constructs might generate additional, uncharacterized siRNAs, our CRISPR/Cas9 edits specifically targeting the predicted siRNA-generating regions in *SUC5* and *SUC8* produced changes in *SUC2* exon 2 methylation and expression that are consistent with those observed in the overexpression lines, and consistent with a role of *SUC5* and *SUC8* as siRNA origins.

It will be exciting to find out if *SUC5* and *SUC8* only serve as siRNA origins, similar to the pseudogenes proposed as origins of other gene-derived siRNAs [54], or if they pertain a function in producing sucrose transporters. Since we find methylation enrichment on the *SUC5* and *SUC8* genes themselves, the siRNAs might play a role in suppressing their transcriptional activity. It should also be noted that, while we focus on *SUC5* and *SUC8* here, the high similarity in sequences and methylation patterns of *SUC6*, *SUC7* and *SUC9* could mean that these genes are also involved in the siRNA-mediated regulation. Furthermore, we cannot exclude a role for siRNAs originating from *SUC2* itself.

The observed correlation between siRNA abundance and *SUC2* methylations, as well as max summer temperature, across accessions, and the observations on plant lines with manipulated siRNA levels or siRNA sequence, led us to hypothesize that a climate-dependent regulation of *SUC5* and *SUC8* could be responsible for the climate-dependent variation in *SUC2* transcript levels. We found this hypothesis corroborated by the positive correlation of *SUC5* and *SUC8* transcript levels with max summer temperatures. However, further experiments aimed at identifying sequence polymorphisms or climate-dependent transcription factors are needed to verify this mechanism. While evaluating the production of specific siRNAs, it should also be considered that the general activity of the RdDM machinery might be influenced by the climatic conditions [59].

Our results establish exon methylation mediated by gene-derived siRNAs as a mechanism for plant adaptation. How the CHH methylation hotspot on the *SUC2* exon came about remains unclear. It has been speculated that one of *SUC2*’s introns is derived from a transposon [60], which might have introduced CHH sites to the gene. Clearly, more work is needed in this regard. Especially important will be finding out if *SUC2*’s methylation pattern is an anomaly among plant genes or if other genes are regulated similarly.

## Materials and methods

### Plant material and growth conditions

Seeds of 1,135 natural accessions (N78942) were obtained from the Eurasian Arabidopsis Stock Centre [61]. T-DNA insertion mutants *suc8-1* (SALK_067656C), *suc8-2* (SALK_118484C), *ros1-4* (SALK_045303C), *rdr2-3* (SALK_206644C), *rdr2-4* (SALK_207201C), *dcl3* (SALK_005512), *ago6* (SALK_031553C), *nrpd1-2* (SALK_057637C), *nrpd1-3* (SALK_128428), *nrpe1-2* (SALK_029919) and *nrpe1-11* (SALK_017795C) were purchased from Arashare (https://www.arashare.cn/index/). The generation of *suc5* mutants, *SUC5:SUC5fr* and *SUC8:SUC8fr* plants is described below. Murashige and Skoog (MS) solid medium was prepared with 1% (w/v) sucrose and 1% (w/v) agar at pH 5.8. After vernalization (4 °C, 3 days, 24 h dark), the seeds were grown on 1/2 MS medium plates for about 5 d (22 °C, 24 h dark) until germinated and then transferred to a growth chamber on moist soil in 7 cm square pots kept under 16-h-light/8-h-dark period, a light level of 200 mmol photons m^−2^ s^−1^ and a temperature of 21 °C. Experiments were conducted on 4-week-old plants unless stated otherwise. Plants grown for transcriptomics and methylomics analysis were grown under the same long day-conditions, but at 22 °C [23]. Their transcriptomics experiments were also conducted on 4-week-old plants.

### Measurement of phloem sugar concentrations

Measurement of phloem exudate sugar levels was adapted from Xu and colleagues [33]. A vacuum concentrator (Eppendorf, Germany) was used to increase the concentration of sugars in the sample. Sugar analysis was then performed on a high-performance liquid chromatography system coupled to a refraction index detector (HPLC-RI, Shimadzu, Japan). A NH_2_ column (4.6 × 250 mm, 5 mm, Shimadzu) was used as separation column at 40 °C with acetonitrile/deionized water, 7:3 (v/v) as mobile phase and a flow rate of 1 mL/min with 20 μL injection volume. The relative concentrations of the different sugars were analyzed quantitatively by the peak area normalizing method.

### Analysis of genomic sequence associations with climatic variables

The GenoCLIM algorithm [16] was used to identify associations between the genetic variation across 1,135 naturally inbred *A. thaliana* accessions and climatic variables. The query region contained the gene of interest as well as the 5 kb upstream of it.

### Measurement of esculin export from leaves

The esculin assays were carried out as described by Ren and colleagues [34]. Esculin (1mM, TCI, Tokyo, Japan) solution was infiltrated through the abaxial epidermis of leaves using a 1 mL-syringe without needle. After 2 h, the leaf area was measured and the leaves transferred into 2 mL tubes to be homogenized in a high-flux tissue grinder (SCIENTZ-48, Ningbo Scientz Biotechnology, Ningbo, China). The samples were incubated for 30 min at 50 °C in 1 mL solution containing 60% methanol, 25% chloroform, and 15% distilled water. Samples were centrifuged for 10 min at 4,000 rpm and the supernatant was transferred into new tubes. The sample was mixed with distilled water of 60% volume of the supernatant and allowed to stand at room temperature for 2 h. Samples were then diluted 10-fold and 200 μL placed into a 350 µL cuvette (ThermoFisher Scientific, CA, USA) to measure fluorescence intensity using a microplate reader (Infinite M200 Pro, Tecan, Männedorf, Switzerland) with excitation at 335 nm and fluorescence detection between 455 and 465 nm. The esculin export was calculated as 1 divided by the difference between fluorescence intensity after labeling *I*_*1*_ and intensity of an unlabeled control *I*_*C*_ divided by leaf area *A*, which translates to the following formula:


$$\begin{array}{c} Esculin\;export\;=\frac{A}{\left(I_{\text{1}}-I_{C}\right)} \end{array}$$

### Analysis of mRNA levels and protein abundance

Total RNA from 100 mg of leaf material was extracted using Trizol (Invitrogen, Carlsbad, CA, USA) and qPCR performed using TransStart Tip Green qPCR SuperMix (Transgen Biotech, Beijing, China) on a CFX Connect Real Time System (Bio-Rad, Germany). Primers are listed in S4 Table. The qPCR experiments were performed on at least three independent biological samples and on three technical replicates, and the Cq values for these replicates were averaged. The mRNA levels of *SUC2* were normalized to mRNA levels the two reference genes *ACT12* (AT3G46520) and *UBQ10* (AT4G05320) mRNA levels and quantified according to the 2^−ΔΔCt^ method [62]. Data on *SUC2* mRNA levels from transcriptomics experiments were obtained for all 656 accessions analyzed by RNA sequencing by Kawakatsu and colleagues [23]. The dataset is listed on Gene Expression Omnibus (GEO) as Dataset GSE80744. Gene expression values had the unit of fragments per kilobase exon per million reads (FPKM).

The anti-SUC2 polyclonal rabbit antibody was raised and affinity-purified against a peptide of SUC2 corresponding to the amino acid residues 308–322 (VYGGNSDATATAASK) and 391–404 (DHGGAKTGPPGNVT) by Wuhan GeneCreate Biological Engineering (Wuhan, China). The final concentration of affinity-purified antibodies used for immunoblot analysis was about 40 ng/mL. Plant-specific Anti-ACTIN rabbit polyclonal antibody, used as reference for western-Blots, was purchased from Sangon Biotech (Beijing, China). Specificity was tested by western Blot, with occurrence of a single band of the correct molecular weight indicating a positive result.

Membrane protein isolation and quantification by Western Blot followed the protocol of Xu and colleagues [9]. Arabidopsis leaf material (1 g) was collected and ground into powder in a high-flux tissue grinder (SCIENTZ-48, Ningbo Scientz Biotechnology, Ningbo, China). The protein was extracted using a buffer containing 50 mM NaH_2_PO_4_, adjusted to pH 8.0 with NaOH, 200 mM NaCl, 1 mM EDTA, 0.6% PVP-30, 5 mM ascorbic acid, 2 mM DTT, 50 mM sodium pyrophosphate, 25 mM sodium fluoride, 1 mM sodium molybdate, 1 mM 1,10-phenanthroline, 5 mM β-sodium glycerin phosphate, 0.5 mM PMSF, dissolved in ethanol and 1× protease inhibitor cocktail. The samples were placed in 2 mL protein extraction buffer and mixed thoroughly, followed by centrifugation for 15 min at 10,000 *g* and 4 °C. The supernatant was transferred into a new tube and the centrifugation repeated. The combined supernatants were centrifuged at 100,000 *g* for 1 h at 4 °C. The supernatant was discarded and the precipitate resuspended in suspension buffer (2 mM EGTA, 2 mM EDTA,100 mM MOPs, adjusted to pH 7.0 with NaOH, 1 mM DTT, 0.5 mM sodium pyrophosphate, 0.5 mM sodium fluoride, 0.5 mM sodium molybdate, 0.5 mM PMSF dissolved in ethanol, 10% glycerol and 1× protease inhibitor cocktail). Protein levels were quantified using the Easy II Protein Quantitative kit (BCA, TransGen Biotech) and a spectrophotometer (Infinite M200Pro, Tecan, Switzerland).

The tracer bromophenol blue was added to 80 μg protein and the sample mixed. To denature the samples, they were heated at 100 °C for 5 min. The SDS-PAGE was run with the 5% stacking gel for 0.5 h at 80 V and the 12% separating gel for approximately 1 h at 120 V using the DYY-6D Protein equipment (Liuyi, Beijing, China). For membrane transfer, Polyvinylidene fluoride (PVDF) membrane and filter papers of the same size as the gel were used. The transfer sandwich was assembled by incubating them in TBST buffer for 10 min and then arranging them in the following order: four filter papers, PVDF membrane, gel, and four filter papers from the positive to negative pole. The semidry electrotransfer was carried out in the JY-ZY3 semidry cell (Junyi Biotechnology, Shanghai, China) at a constant current of 70 mA for 85 min at room temperature until the gel’s proteins were thoroughly transferred to the PVDF membrane. The PVDF membrane was washed with TBST buffer for 5 min three times and subsequently blocked with 5% skim milk dissolved in TBST buffer for 1.5 h at room temperature. Following this, the PVDF membrane was incubated with SUC2-specific polyclonal rabbit antibody described in Xu and colleagues [8] for 1.5 h, and then with the secondary antibody (Goat anti-Rabbit IgG, HRP conjugated, diluted 5,000-fold in 5% skim milk in TBST) for 2 h on a shaker. The membrane was washed with TBST buffer for 5 min three times after each incubation. Finally, the protein bands were imaged using the EasySee western blot kit (TransGen Biotech) with the Molecular Imager Gel DOC XR+ Imaging System (Bio-Rad, Germany). The protein abundance was quantified using ImageJ [63].

### Analysis of epigenetic marks

Epigenetic marks on the genes and promoter regions (1.5 kb upstream of coding sequence) were investigated using the 1001 *Arabidopsis* Methylomes map [23]. Gene and promoter sequences from 30 accessions, selected to represent the full range of summer temperature (S1 Table; S2 Fig), were aligned and a high-resolution screenshot was taken. These were then used to count epigenetic marks in each accession. Epigenetic marks on SUC family genes in the Col-0 accession were further analyzed with the help of the AraENCODE database [64].

Chop-qPCR as described by Zhang and colleagues [65] was used to quantify methylation of the *SUC2* gene. Genomic DNA was extracted from 100 mg leaf tissue and treated with RNase A. DNA (1 µg) was digested with the *HaeIII* endonuclease in a 50 μL reaction mixture. After overnight incubation at 37 °C, *SUC2* DNA methylation levels were quantified by qPCR as described above. The undigested template was used as a control. Methylations protect DNA from digestion by *HaeIII*, thereby influencing the abundance of the qPCR product.

### Partial Mantel test and linear regression analysis

Partial Mantel tests and linear regression analysis were performed in R (version 4.5.1). Partial Mantel tests were conducted with the number of *SUC2* CHH/CG/CH methylations coded as dependent variables and max summer temperatures coded as independent variables. To control for the confounding effect of genetic structure, a genetic distance matrix was constructed based on the first five principal components (PC1-5) derived from 50,000 randomly selected SNPs. This matrix was used as a covariate in the partial Mantel test. Prior to the analyses, the variables were transformed to Euclidian distance matrices.

For linear regression analysis, *SUC2* CHH/CG/CH methylations were set as dependent variables while max summer temperature was the independent variables. The principal components (PC1-5), derived from 50,000 randomly selected genome-wide SNPs, were included as covariates to control for population structure. The analysis was performed using the lm function in R.

### Manipulation of DNA methylation

For the removal of methylations, vernalized seeds were sown on filter paper in petri dishes containing 50 μmol 5-azacytidine (5-Aza; Sigma Aldrich, USA) dissolved in 1.4 ml water, following the protocol of Xie and colleagues [29]. Seeds were kept at 22 °C with 16 h/8 h light-dark-cycle until germinated and then transferred to soil in 7 cm square pots. Plants were grown under 21 °C, 16 h-light/8 h-dark (200 mmol photons m^−2^ s^−1^) conditions for 4 weeks before DNA extraction and chop-qPCR.

### Identification of siRNAs

To identify siRNAs targeting exon 2 of *SUC2*, its sequence was screened against a database of *A. thaliana* small RNAs [66], which provides the number of reads for siRNAs binding to a certain site. DsRNA sequence data from floral bud tissue, obtained from Li and colleagues [67] (GEO accession GSE57215), was used for the identification of the precursor siRNA sequence. This dataset was generated on *dcl234* mutant plants, which lack proteins to process siRNA precursors and, thereby, leads to their accumulation. Sequencing reads of the siRNA1 precursor were found through BLAST search using the siRNA sequence as query. Data on siRNA abundance in Col0 control and T-DNA-insertion mutants of RNA polymerase IV (*nrpd1-4*, SALK_083051) and V (*nrpe1-12*, SALK_033852) were obtained from Liu and colleagues [68] (GEO accession GSE100010), Li and colleagues [67] (GSE57215), and Lee and colleagues [69] (GSE36424). Processed data were downloaded and imported into the Integrated Genome Browser [70], where reads per million were quantified at the siRNA origin (Chromosome 2, 6,274,938–6,274,962).

### Quantification of siRNA abundance

Stem-loop RT-qPCR, following the procedure described by Zhang and colleagues [65], was used to quantify the abundance of siRNAs. RNA was extracted from 100 mg flower tissue using the Trizol (Invitrogen) method. Total RNA (1 μg) was digested with RNase-free DNase to erase genomic DNA and reverse transcribed into complementary DNA using a siRNA-specific reverse transcription primer (S4 Table), followed by PrimeScript RT reagent kit with gDNA eraser (Takara Bio, Japan). The mix was incubated at 42 °C for 2 min followed by 37 °C, 15 min and 5 sec at 85 °C. qPCR was carried out using the 2X TransStart Tip Green qPCR SuperMix (Transgen) on a CFX Connect Real Time System (Bio-Rad). As recommended by Zhang and colleagues [65], chromosome associated kinesin *snoR101* (AT1G05563) was used as an internal control.

### Genome wide association study

GWAS was performed using easyGWAS [71] and the 1001 Genomes dataset with Tair10 gene annotations. The number of CHH methylations on the *SUC2* exon 2 in 419 accessions was used as dependent variable. The EMMAX algorithm [72] was used without applying a minor allele frequency filter. A correlation was deemed significant when the *p*-value was lower than the Bonferroni threshold at *p* = 0.00005.

### Plasmid construction and plant transformation

*SUC5* (−1,611 ~ 1,410 bp), *SUC8* (−1,545 ~ 1,390 bp), and SUC8-edited (−1,545 ~ 1,390 bp) gene sequences were amplified with gene-specific primers (S4 Table). The purified PCR fragment was constructed into PBI121 vector through *ScaI* and *SacI*. For *SUC5* CRISPR/Cas9 construction, two specific target gRNAs primers were designed by CRISPR-P 2.0 (http://crispr.hzau.edu.cn/CRISPR2/). PCR fragment was amplified from pCBC-DT1T2 and was cloned into binary vector pKSE401 after purification, using Golden gate method as described in Xing and colleagues [73]. Then recombinant plasmid was transformed into *Agrobacterium tumefaciens* strain GV3101. Ecotype IP-Ver-5 and Castelfed-1-197 were used as background to generate transgenic lines through a floral dipping method [74]. Half-strength Murashige and Skoog medium with 1% sucrose, 1% agar, and 50 mg/mL kanamycin was used to screen positive transformants.

### Accession numbers

Relevant information on the genes used in this study can be found under the gene IDs AT1G71880 (*SUC1*), AT1G22710 (*SUC2*), AT2G02860 (*SUC3*), AT1G09960 (*SUC4*), AT1G71890 (*SUC5*), AT5G43610 (*SUC6*), AT1G66570 (*SUC7*), AT2G14670 (*SUC8*), AT5G06170 (*SUC9*), AT2G36490 (*ROS1*), AT4G11130 (*RDR2*), AT3G43920 (*DCL3*), AT2G32940 (*AGO6*), AT1G63020 (*NRPD1*), and AT2G40030 (*NRPE1*)

## Supporting information

S1 Table Natural accessions used for different experiments. Accessions were randomly selected from the available stocks to represent a broad range of max summer temperatures. Summer temperatures were derived from the WorldClim v.2 dataset.
(XLSX)

S2 Table Pearson correlation of maximal summer temperature with other climatic variables of the WoldClim v2 dataset. Correlations with *P*-Value below the Bonferroni-limit of 8e–05 were considered significant.
(XLSX)

S3 Table SNP annotations of loci with significant association between genetic variation and number of methylations on *SUC2* exon 2 according to GWAS. Correlations with *P*-Value below the Bonferroni-limit of 1e–05 were considered significant.
(XLSX)

S4 Table List of primers used in this study. (XLSX)

S1 Fig Indicators for phloem loading rates and geographic locations of *A. thaliana* accessions. **A)** Phloem exudate sucrose content. **B)** Esculin fluorescence intensity in arbitrary units (AU). Measurements were performed on source leaves of 4-weeks-old plants, all grown under the same controlled conditions. The numbers above the bars/boxes indicate the maximal summer temperature at the accessions’ natural habitats. The temperatures below 25 °C are indicated by blue shading, while temperatures above 25 °C are indicated by gray shading. **C)** Correlation of esculin fluorescence with exudate sugar content. Higher values of the leaf esculin fluorescence measured here indicate a lower esculin export rate. The diamond markers represent the group means for both esculin fluorescence and sugar content. Regression line indicates significant Spearman’s rank correlation (*P* < 0.05). **D)** Original geographic distribution of *Arabidopsis thaliana* accessions used in Fig 1A–1D. The map was generated using R (version 4.6.0) based on data from Natural Earth (https://www.naturalearthdata.com), which is in the public domain. In box plots, average values are indicated by lines, quartiles by box. Error bars indicate standard deviation. Number of biological replicates per accession: 5 (A), ≥7 (B), ≥9 per group (C). The data and script underlying this figure can be found in the supplemental information file S2 Data.
(TIF)

S2 Fig *SUC2* methylation in 30 natural accessions and association of CHH/CG/CH methylations with max summer temperature. **A)** Screenshot from the 1,001 epigenome browser. Accessions were arranged according to the max summer temperature in their natural habitat, with lowest temperatures at the top and highest temperatures at the bottom. Accessions were chosen to represent the full range of max summer temperatures. Col-0 does not show methylations at exon 2, which might be an artifact considering that many other methylomics approaches measured exon 2 methylations in that accession (see, e.g., S6 Fig). **B)** Visualization of partial Mantel test of *SUC2* CHH/CG/CH methylations with max summer temperature across 419 accessions. Error bars represent 95% confidence intervals. **C)** Visualization of linear regression analysis of *SUC2* CHH/CG/CH methylations with max summer temperature across 419 accessions. Regression line indicates significant correlation (*P* < 0.05). ns: *p* > 0.05, ** *p* < 0.01. The data and script underlying this figure can be found in the supplemental information file S2 Data.
(TIF)

S3 Fig Information on RdDM-related mutants used in this study. **A)** Illustration of the RdDM pathway genes and their modification through T-DNA insertions in the *ros1*, *rdr2*, *dcl3*, *ago6*, *nrpd1*, and *nrpe1* mutant lines. **B)** *SUC2* mRNA level in the *ros1*, *rdr2* mutant lines. **C)** *SUC2* mRNA level in the Col-0 wild-type and *dcl3*, *ago6*, *nrpd1*, and *nrpe1* mutant lines. **D)** *SUC2* mRNA level in the *cmt3*, *cmt2cmt3*, *met1* and *drm1drm2cmt2cmt3* mutant lines. **E)** CHH methylations on the whole *SUC2* gene body and on *SUC2* exon 2 in Col-0 wild-type and *met1* mutant relative to the total number of CHH sites within the regions. **F)** CHH methylation reads in Col-0 wild-type and *met1* mutant. Samples in B, C were source leaves of 4-week-old plants grown under controlled long day-conditions at 21 °C and analyzed by qPCR. RNA sequencing data in D were obtained from GEO datasets: GSE183985 (source leaves from 5-week-old plants grown on soil under controlled long day-conditions at 22 °C), GSE22957 (source leaves from 4-week-old plants grown on soil under controlled long day-conditions at 22 °C) and GSE247353 (10-day-old seedlings grown on MS agar medium under controlled long day-conditions at 22 °C). The CHH methylation data in E were obtained from bisulfite sequencing datasets GSE122394 (2-week-old seedling grown on plates under continuous light at 22 °C) and GSE181896 (source leaves of 4-week-old plants grown under long day-conditions at 22 °C). The methylation reads in F were extracted from GSE181896, too. Bars indicate standard deviation. Different letters indicate significant differences according to *t* test (*P* < 0.05). In B–D: *N* = 3 (*ros1*, *rdr2, met1, drm1drm2cmt2cmt3*), 6 (*dcl3*, *ago6*, *nrpd1*, and *nrpe1*), 2 (*cmt3*, *cmt2cmt3*); *N* = 2 (E), ≥10 (F). The data and script underlying this figure can be found in the supplemental information file S2 Data.
(TIF)

S4 Fig siRNA candidates for binding of the *SUC2* gene according to the Plant massively parallel signature sequencing (MPSS) database. **A)** Illustration of *SUC2* gene (blue) with indicated positions of potential short RNA binding events and siRNA length (orange, pink, green, light blue, and gray marks). Arrow indicates *SUC2*’s exon 2. **B)** Illustration indicating number of experiments in which siRNA candidates were identified for regions of the SUC2 gene. The highest bar corresponds to *SUC2*’s exon 2 (arrow).
(TIF)

S5 Fig Information on genetically modified plant lines used in this study. **A)** Illustration of *SUC5* gene sequence modified by CRISPR/CAS9-technology in the *suc5-2* and *suc5-3* mutant lines. The blue triangle indicates the origin location of siRNA1, and the red lines (F1, F2) represent the start and end points of the amplified fragment of the *SUC5:SUC5fr* overexpression lines. **B)** Illustration of the *SUC8* gene modified through T-DNA insertion in the *suc8-1* and *suc8-2* mutant lines. The blue triangle indicates the origin location of siRNA2, and the red lines (F3, F4) represent the start and end points of the amplified fragments of the *SUC8:SUC8fr* overexpression lines. **C)** *SUC2* mRNA level in wild-type and *SUC5:SUC5fr* lines. **D)** *SUC2* mRNA level in wild-type and *SUC8:SUC8fr* lines. **E)** Esculin fluorescence of 4-week-old Arabidopsis leaves in wild-type and *SUC5:SUC5fr* lines. **F)** Esculin fluorescence of 4-week-old Arabidopsis leaves in wild-type and *SUC8:SUC8fr* lines. **G)** *SUC2* mRNA level in wild-type and *suc5* mutants. **H)** *SUC2* mRNA level in wild-type and *suc8* mutants. **I)** Esculin fluorescence of 4-week-old Arabidopsis leaves in wild-type and *suc5* mutants. **J)** Esculin fluorescence of 4-week-old Arabidopsis leaves in wild-type and *suc8* mutants. **K)** Illustration of the bases that were edited in the siRNA2 sequence in transgenic *SUC8:SUC8fr*-edited lines. **L)** *SUC2* mRNA level in IP-Ver-5, *SUC8:SUC8fr-2* and *SUC8:SUC8fr*-edited lines. Error bars indicate standard deviation. Different letters indicate significant differences according to *t* test (*P* < 0.05). In box plots, average values are indicated by lines, quartiles by box. Number of biological replicates: 4 (C, G), 3 (D, H, and L), ≥9 (E, F), ≥6 (I, J). The data underlying this figure can be found in the supplemental information file S2 Data.
(TIF)

S6 Fig Illustrations of genomic structure and DNA methylations of Arabidopsis SUCROSE TRANSPORTERs (SUCs) in accession Col-0. The bright green rectangle highlights exon 2-methylations. The information was obtained from the AraENCODE database.
(TIF)

S1 Data Data underlying all diagrams of the main manuscript. (XLSX)

S2 Data Data underlying all diagrams of the supporting information figures. (XLSX)

S1 File Collection of R scripts that were used to generate figures of the main manuscript and the supporting information. (ZIP)

## Abbreviations

5-Aza: 5-aza-2′-de-oxycytosine
AGO6: argonaute6
AU: massively parallel signature sequencing
DCL3: dicerlike3
FPKM: fragments per kilobase exon per million reads
GEO: gene expression omnibus
GWAS: genome wide association study
MPSS: massively parallel signature sequencing
MS: Murashige and Skoog
NRPD1: nuclear RNA polymerase D1
NRPE1: nuclear RNA polymerase E1
PVDF: polyvinylidene fluoride
RdDM: RNA-directed DNA methylation
RDR2: RNA-dependent RNA polymerase 2
ROS1: repressor of silencing1
siRNA: short interfering RNA
SUC2: sucrose transporter 2.
