# Supplementary material for: siRNA-guided DNA-methylation at a sucrose transporter gene potentially contributes to climatic plant adaptation
Source: PLoS Biol. 2026 Jul 30;24(7):e3003919. doi: 10.1371/journal.pbio.3003919 (PMC13440838; doi:10.1371/journal.pbio.3003919)
Supplement: Supplementary file 1 [file pbio.3003919.s016.docx]

Dear Editor,

please find a detailed response to the comments below.

Comment 2, Population structure: The correlation now is only about half what it was before (R 0.06 instead of 0.12), although it is still significant. But why do the authors no longer provide a comparison with the partial correlation coefficient of summer temperature with CHH methylation (which was 0.01 before)?

Regarding the lower R: In our original submission, the phylogenetic relatedness matrix was generated from a single nuclear DNA marker (PepC). In response to the Editor’s comments, we adopted a more rigorous approach: a genetic distance matrix derived from the first five principal components of 50,000 randomly selected SNPs. This genome-wide method provides a more comprehensive control for population structure.

The partial Mantel test shows that summer temperature remains significantly correlated with CHH methylation after controlling for population structure (R = 0.056, P = 0.0048). The reduction of the R value from 0.123 (with PepC) to 0.056 (with SNPs) is expected because the SNP-based method removes more variance attributable to true population structure, leaving a smaller but more accurate estimate of the temperature-specific signal.

Thank you for pointing out the omission of information regarding the potential partial correlation of population structure with CHH methylation. We checked this value in our new partial Mantel analysis. After controlling for temperature, population structure is not significantly correlated with CHH methylation (R = 0.021, P = 0.213). This result is similar to the original analysis. We included the information in the results section:

Line 169 – 172: “The partial Mantel test revealed a significant correlation of summer temperature with CHH methylations (R = 0.056; P = 0.0048) (Fig. S2B). The correlation of population structure with CHH methylations was not significant (R = 0.021, P = 0.213).”

Comment 3, regarding the small effect sizes of RdDM mutations: I find the response largely circular and hand-wavy. It can be interpreted as “small RNAs aren’t so important after all”. The authors should try harder to address my concern.

Rechecking our data, we discovered that biological replicates were incorrectly specified in part of the statistical analysis. The updated analysis shows that *SUC2* mRNA levels are significantly increased in 7 out of 8 RdDM mutants (not 5 out of 8 as written previously). The corresponding sentence in the results section was revised (line 217) and the data separated in two panels as Fig. S3B and C.

Nevertheless, the small effect size relative to transcript level variation among natural accessions remains a valid concern. You are right to remind us of the importance of methylations on mRNA levels demonstrated in other parts of the manuscript. Most relevant in this context are the 5-Aza experiments. Like the RdDM-mutants, 5-Aza treatment indiscriminately affects methylations on all genes, not only on *SUC2*. Moreover, both types of experiments were conducted under the same controlled conditions.

We checked the 5-Aza treatment effect specifically for Col-0, the background of the RdDM mutants. *SUC2* mRNA levels roughly doubled in the treated plants relative to the control. This represents a stronger effect than observed in most RdDM mutants.

One principal difference between the experiments is that RdDM pathway mutations specifically act on CHH methylations, while 5-Aza treatment affects methylations in all contexts. While we show that CG and CH methylations do not correlate with max summer temperature when corrected for population structure (Fig. S2B, C), an influence of CG and CH methylations specifically in accessions from habitats with high summer temperatures remains a possibility.

We included additional experimental data to check if hypothesizing an effect of CG or CH methylations on CHH methylations is sensible (Fig. S3D-F). The analysis of bisulfite sequencing data from mutants deficient in CH methylations does not provide any indication of cross-talk between CH and CHH. The situation is different for the CG-deficient *met1* mutant, where CHH methylations on *SUC2*’s exon2 trended down and *SUC2* mRNA levels trended up. Together with the varying presence of CG marks on *SUC2* exon 2 across accessions, and the published work on the crosstalk between the two types of marks (e.g. Srikant et al. 2022, 10.1186/s13059-022-02833-5), a potential relevance of CG marks in the present context should not be excluded.

We added the following text to the discussion to point out the issue of effect size in RdDM pathway mutants and its potential meaning: “Moreover, CHH methylations might also be influenced by CG methylations ^50^, which showed a presence on *SUC2*’s exon 2 that varied across accessions. The smaller effect on *SUC2* mRNA levels in plants with disrupted RdDM pathway compared to in plants where all methylations were removed by 5-Aza treatment could be due to a contributing role of CG methylations.” (Lines 355 – 359).

Data on SUC2 transcript and methylation levels were added to the supplemental material as Fig. S3D-F. The figure legend to Fig. S3 was updated accordingly. Moreover, in the results part, we write:

“To test if CG or CH methylations could play a role, we checked *SUC2* transcript levels in mutants that cannot establish CH methylations (*cmt3, cmt2cmt3*) or CG methylations (*met1*), while maintaining an intact RdDM pathway^37,38^. *SUC2* transcripts levels similar to the Col-0 wild-type were detected in *cmt2* and *cmt2cmt3* mutants (Fig. S3D). Only with additional disruption of the RdDM pathway in the *drm1drm2cmt2cmt3* mutant did *SUC2* transcript levels increase (Fig. S3D). Interestingly, transcript levels in the CG-less *met1* mutants trended up, although without reaching the significance threshold (p=0.098; Fig. S3D). Analysis of methylations showed the loss of CG methylations in *met1* coinciding with the CHH methylation rate and on *SUC2*’s exon2 trending down and CHH reads being reduced by about 40% in source leaves (Fig. S3E,F).” (Lines 221-231)

Comment 4, regarding chop-PCR: The authors admit that I am correct and then say that they cannot do anything about this. However, adding the qualifying statement they propose is acceptable.

Thank you for your understanding.
