# Supplementary material for: siRNA-guided DNA-methylation at a sucrose transporter gene potentially contributes to climatic plant adaptation
Source: PLoS Biol. 2026 Jul 30;24(7):e3003919. doi: 10.1371/journal.pbio.3003919 (PMC13440838; doi:10.1371/journal.pbio.3003919)
Supplement: Supplementary file 2 [file pbio.3003919.s017.docx]

IMPORTANT - please attend to the following:

a) Overall, the Academic Editor (AE) is satisfied but have suggested several modification (i.e., change the existing wording to) that improve clarity in different sections of the manuscript (see the foot of this letter). Please attend to these test modifications from the Academic Editor.

**Thank you for the suggestions. We have implemented them as detailed below.**

b) Please address my Data Policy requests below; specifically, we need you to supply the numerical values underlying Figs 1ABCD, 2ABCDEF, 3BCDE, 4CEFHIKLNOP, 5ABC, S1ABC, S2BC, S3BCDEF, S5CDEFGHIJL, S6ABCDEFG either as a supplementary data file or as a permanent DOI’d deposition (such as Zenodo).

**The numerical data underlying the figures has been assembled in two xlsx-files, one for main manuscript figures and one for supplemental figures. These files are submitted as supplemental data. No numerical values are available for Fig. S6, as this shows screenshots of the AraENCODE database.**

c) Please clearly cite the location of the data in all relevant main and supplementary figure legends, for example: “The data underlying this figure can be found at doi.org/10.xxx/zenodo.xxxxx.” We understand that this may appear repetitive, but it helps make the figures and their legends more self contained.

**We decided to attach all data directly to the article in xslx-files. Accordingly, we write in figure legends: ”The data underlying this figure can be found in the supplemental information file S1_Data.” Or for the supplemental figures: “The data and script underlying this figure can be found in the supplemental information file S2_Data.”**

d) Please include the URLs of your funders in the Financial Disclosure statement.

**We added the URLs (**[**https://www.nsfc.gov.cn/**](https://www.nsfc.gov.cn/) **and https://kjt.shaanxi.gov.cn) on Line 627-628.**

e) Please make any custom code available, either as a supplementary file or as part of your data deposition.

**The R code used to make figures S1CD, S2BC, S3CDE, including underlying data, were prepared. It is attached in a zip-file for the supplemental material.**

f). "Significance Statement" - we don't do these at PLOS Biology. So, please delete it.

**Significant Statement (Line50-60) has been deleted.**

**Suggested modifications by AE (Lightly Edited)**

**Title: Change the title to:**

siRNA-guided DNA methylation at a sucrose transporter gene potentially contributes to climatic adaptation in plants

**Abstract:**

Replace “Exploration of methylome data and epigenome editing experiments indicated that epigenetic marks on SUC2’s exon 2 could enact the climate-dependent expression of SUC2.” with “Exploration of methylome data and epigenome editing experiments suggested that epigenetic marks on SUC2’s exon 2 contributed to the climate-dependent expression of SUC2.”

Replace “Besides illustrating a novel regulatory strategy of carbon export from leaves, our results establish exon methylation mediated by gene-derived siRNAs as a mechanism for plant adaptation.” with “Besides illustrating a novel regulatory strategy of carbon export from leaves, our results establish exon methylation mediated by gene-derived siRNAs as yet another potential mechanism for plant adaptation.”

**The title and abstract have been modified as AE suggested. The changed text was marked in purple in the track-changes version of the manuscript.**

**Results:**

Line227-228:

Instead of “Interestingly …” write “met1 mutants lack all CG methylation, which was accompanied by fewer CHH reads in source leaves and less CHH methylation on exon 2 of SUC2, although there was not a robust effect on SUC1 expression (p=0.098).”

**The text （Line 228-232）has been deleted and replaced as “met1 mutants lack all CG methylation, which was accompanied by fewer CHH reads in source leaves and less CHH methylation on exon 2 of *SUC2*, although there was not a robust effect on *SUC2* expression (p=0.098) (Fig. S3D-F).” Line 232-235**

As you address these items, please take this last chance to review your reference list to ensure that it is complete and correct. If you have cited papers that have been retracted, please include the rationale for doing so in the manuscript text, or remove these references and replace them with relevant current references. Any changes to the reference list should be mentioned in the cover letter that accompanies your revised manuscript.

**All references have been reviewed and found to be correct.**

**Additional revisions**

Regarding figures

**Individual datapoints were plotted in the bar charts of Fig. 4 and Fig. S3.**

Regarding funding statement

i). State what role the funders took in the study. If the funders had no role in your study, please state: “The funders had no role in study design, data collection and analysis, decision to publish, or preparation of the manuscript.”

**The statement is present in lines 628-629.**

ii). If any authors received a salary from any of your funders, please state which authors and which funders.

**The statement is present in lines 629-630.**

2) In your Data Availability Statement, please ensure you have provided all necessary access information in complete sentences. Access information should include the name of any data repositories used as well as digital object identifiers (DOIs), accession numbers or codes, or other persistent identifiers for all relevant data. If all data has been provided within your submission files, please use the statement "All relevant data is available within the manuscript and Supporting Information files." The Data Availability Statement will appear on the published version of your manuscript.

The statement was updated to reflect that all data and code are available as part of this publication.

4) Some material included in your submission may be copyrighted. According to PLOS’s copyright policy, authors who use figures or other material (e.g., graphics, clipart, maps) from another author or copyright holder must demonstrate or obtain permission to publish this material under the Creative Commons Attribution 4.0 International (CC BY 4.0) License used by PLOS journals. Please closely review the details of PLOS’s copyright requirements here: PLOS Licenses and Copyright. If you need to request permissions from a copyright holder, you may use PLOS's Copyright Content Permission form.

Please respond directly to this email and provide any known details concerning your material's license terms and permissions required for reuse, even if you have not yet obtained copyright permissions or are unsure of your material's copyright compatibility. Once you have responded and addressed all other outstanding technical requirements, you may resubmit your manuscript within Editorial Manager.

Potential Copyright Issues:

[Figure S1D]: please (a) provide a direct link to the base layer of the map (i.e., the country or region border shape) and ensure this is also included in the figure legend; and (b) provide a link to the terms of use / license information for the base layer image or shapefile. We cannot publish proprietary or copyrighted maps (e.g. Google Maps, Mapquest) and the terms of use for your map base layer must be compatible with our CC BY 4.0 license.

Note: if you created the map in a software program like R or ArcGIS, please locate and indicate the source of the basemap shapefile onto which data has been plotted.

**We used R to generate the map of Figure S1D. The package was updated to meet the requirement of PLOS Biology. In the supplemental Information, figure legend to Fig. S1D, we explain: “****The map was generated using R (version 4.6.0) based on data from Natural Earth (https://www.naturalearthdata.com), which is in the public domain.”**

Reviewing figures and original data enabled us to spot the following inaccuracies that were revised:

1. The y-axis title of figure S3D was corrected from “SUC2 mRNA level (2−ΔΔCT)” to “SUC2 mRNA level (FPKM)”. The legend was updated to explain the abbreviation.

2. A dataset label in the figure legend to figure S3 was mixed up. It was corrected from “GSE181896” into “GSE247353”. Moreover, plant age was corrected from “12-day-old seedlings” to “10-day-old seedlings”.

3. The number of biological replicates in Fig. 4 was corrected to 4. Individual datapoints were plotted.

4. The number of accessions used for GWAS analysis was corrected to 419 (same as in Fig. 2C). Previously, it said 1135 in the results and 358 in the methods. The data for the 419 accessions in provided in the supplemental data file (S1_Data).
