# Supplementary material for: siRNA-guided DNA-methylation at a sucrose transporter gene potentially contributes to climatic plant adaptation
Source: PLoS Biol. 2026 Jul 30;24(7):e3003919. doi: 10.1371/journal.pbio.3003919 (PMC13440838; doi:10.1371/journal.pbio.3003919)
Supplement: Peer review history

# Editor report: Decision Letter 0

**DOI:** 10.1371/journal.pbio.3003919.r001

**Authors:** Ankiit Ahluwalia

20 Feb 2026

Dear Dr Liesche,

Thank you for submitting your manuscript entitled "siRNA-guided on-exon DNA-methylations on the phloem-loading sucrose transporter contribute to plant adaptation" for consideration as a Research Article by PLOS Biology.

Your manuscript has now been evaluated by the PLOS Biology editorial staff, as well as by an academic editor with relevant expertise, and I am writing to let you know that we would like to send your submission out for external peer review.

However, before we can send your manuscript to reviewers, we need you to complete your submission by providing the metadata that is required for full assessment. To this end, please login to Editorial Manager where you will find the paper in the 'Submissions Needing Revisions' folder on your homepage. Please click 'Revise Submission' from the Action Links and complete all additional questions in the submission questionnaire.

Once your full submission is complete, your paper will undergo a series of checks in preparation for peer review. After your manuscript has passed the checks it will be sent out for review. To provide the metadata for your submission, please Login to Editorial Manager (https://www.editorialmanager.com/pbiology) within two working days, i.e. by Feb 24 2026 11:59PM.

If your manuscript has been previously peer-reviewed at another journal, PLOS Biology is willing to work with those reviews in order to avoid re-starting the process. Submission of the previous reviews is entirely optional and our ability to use them effectively will depend on the willingness of the previous journal to confirm the content of the reports and share the reviewer identities. Please note that we reserve the right to invite additional reviewers if we consider that additional/independent reviewers are needed, although we aim to avoid this as far as possible. In our experience, working with previous reviews does save time.

If you would like us to consider previous reviewer reports, please edit your cover letter to let us know and include the name of the journal where the work was previously considered and the manuscript ID it was given. In addition, please upload a response to the reviews as a 'Prior Peer Review' file type, which should include the reports in full and a point-by-point reply detailing how you have or plan to address the reviewers' concerns.

During the process of completing your manuscript submission, you will be invited to opt-in to posting your pre-review manuscript as a bioRxiv preprint. Visit http://journals.plos.org/plosbiology/s/preprints for full details. If you consent to posting your current manuscript as a preprint, please upload a single Preprint PDF.

Feel free to email us at plosbiology@plos.org if you have any queries relating to your submission.

Kind regards,

Ankiit

Ankiit Ahluwalia,

PLOS Biology

aahluwalia@plos.org

# Editor report: Decision Letter 1

**DOI:** 10.1371/journal.pbio.3003919.r002

**Authors:** Ankiit Ahluwalia

28 Apr 2026

Dear Dr Liesche,

As mentioned in my recent email, this decision is being sent as a formality to formalise the ongoing editorial process. Thank you for your patience while your manuscript "siRNA-guided on-exon DNA-methylations on the phloem-loading sucrose transporter contribute to plant adaptation" was considered at PLOS Biology. Your manuscript has now been evaluated by the PLOS Biology editors and the Academic Editor.

In light of the comments from the Academic Editor, which you will find at the end of this email (and which you have already previously seen), we are pleased to offer you the opportunity to address the remaining points from the Academic Editor in a revision that we anticipate should not take you very long. We will then assess your revised manuscript and your response to the reviewers' comments with our Academic Editor aiming to avoid further rounds of peer-review, depending on the nature of the revisions.

In addition to these revisions, you may need to complete some formatting changes, which you will receive in a follow up email. A member of our team will be in touch with a set of requests shortly. If you do not receive a separate email within a few days, please assume that checks have been completed, and no additional changes are required.

We expect to receive your revised manuscript within 1 month. Please email us (plosbiology@plos.org) if you have any questions or concerns, or would like to request an extension.

At this stage, your manuscript remains formally under active consideration at our journal; please notify us by email if you do not intend to submit a revision so that we withdraw the manuscript.

**IMPORTANT - SUBMITTING YOUR REVISION**

Your revisions should address the specific points made by each reviewer. Please submit the following files along with your revised manuscript:

1. A 'Response to Reviewers' file - this should detail your responses to the editorial requests, present a point-by-point response to all of the reviewers' comments, and indicate the changes made to the manuscript.

*NOTE: In your point-by-point response to the reviewers, please provide the full context of each review. Do not selectively quote paragraphs or sentences to reply to. The entire set of reviewer comments should be present in full and each specific point should be responded to individually.

You should also cite any additional relevant literature that has been published since the original submission and mention any additional citations in your response.

2. In addition to a clean copy of the manuscript, please also upload a 'track-changes' version of your manuscript that specifies the edits made. This should be uploaded as a "Revised Article with Changes Highlighted " file type.

*Resubmission Checklist*

When you are ready to resubmit your revised manuscript, please refer to this resubmission checklist: https://plos.io/Biology_Checklist

To submit a revised version of your manuscript, please go to https://www.editorialmanager.com/pbiology/ and log in as an Author. Click the link labelled 'Submissions Needing Revision' where you will find your submission record.

Please make sure to read the following important policies and guidelines while preparing your revision:

*Published Peer Review*

Please note while forming your response, if your article is accepted, you may have the opportunity to make the peer review history publicly available. The record will include editor decision letters (with reviews) and your responses to reviewer comments. If eligible, we will contact you to opt in or out. Please see here for more details:

https://blogs.plos.org/plos/2019/05/plos-journals-now-open-for-published-peer-review/

*PLOS Data Policy*

Please note that as a condition of publication PLOS' data policy (http://journals.plos.org/plosbiology/s/data-availability) requires that you make available all data used to draw the conclusions arrived at in your manuscript. If you have not already done so, you must include any data used in your manuscript either in appropriate repositories, within the body of the manuscript, or as supporting information (N.B. this includes any numerical values that were used to generate graphs, histograms etc.). For an example see here: http://www.plosbiology.org/article/info%3Adoi%2F10.1371%2Fjournal.pbio.1001908#s5

*Blot and Gel Data Policy*

We require the original, uncropped and minimally adjusted images supporting all blot and gel results reported in an article's figures or Supporting Information files. We will require these files before a manuscript can be accepted so please prepare them now, if you have not already uploaded them. Please carefully read our guidelines for how to prepare and upload this data: https://journals.plos.org/plosbiology/s/figures#loc-blot-and-gel-reporting-requirements

*Protocols deposition*

To enhance the reproducibility of your results, we recommend that if applicable you deposit your laboratory protocols in protocols.io, where a protocol can be assigned its own identifier (DOI) such that it can be cited independently in the future. Additionally, PLOS ONE offers an option for publishing peer-reviewed Lab Protocol articles, which describe protocols hosted on protocols.io. Read more information on sharing protocols at https://plos.org/protocols?utm_medium=editorial-email&utm_source=authorletters&utm_campaign=protocols

Thank you again for your submission to our journal. We hope that our editorial process has been constructive thus far, and we welcome your feedback at any time. Please don't hesitate to contact us if you have any questions or comments.

Sincerely,

Ankiit

Ankiit Ahluwalia,

PLOS Biology

aahluwalia@plos.org

----------------------------------------------------------------

COMMENTS FROM THE ACADEMIC EDITOR:

Comment 2, Population structure: The correlation now is only about half what it was before (R 0.06 instead of 0.12), although it is still significant. But why do the authors no longer provide a comparison with the partial correlation coefficient of summer temperature with CHH methylation (which was 0.01 before)?

Comment 3, regarding the small effect sizes of RdDM mutations: I find the response largely circular and hand-wavy. It can be interpreted as “small RNAs aren’t so important after all”. The authors should try harder to address my concern.

Comment 4, regarding chop-PCR: The authors admit that I am correct and then say that they cannot do anything about this. However, adding the qualifying statement they propose is acceptable.

The responses to my comments 1, 5, 6 all work for me.

# Author comment: Author response to Decision Letter 2

**DOI:** 10.1371/journal.pbio.3003919.r003

26 May 2026

Attachment Submitted filename: Reply_2.docx

# Editor report: Decision Letter 2

**DOI:** 10.1371/journal.pbio.3003919.r004

**Authors:** Ankiit Ahluwalia

18 Jun 2026

Dear Dr Liesche,

Thank you for your patience while we considered your revised manuscript "siRNA-guided on-exon DNA-methylations on the phloem-loading sucrose transporter contribute to plant adaptation" for publication as a Research Article at PLOS Biology. This revised version of your manuscript has been evaluated by the PLOS Biology editors and the Academic Editor.

Based on our Academic Editor's assessment of your revision, we are likely to accept this manuscript for publication, provided you satisfactorily address the remaining points raised by the Academic Editor. Please also make sure to address the following data and other policy-related requests.

IMPORTANT - please attend to the following:

a) Overall, the Academic Editor (AE) is satisfied but have suggested several modification (i.e., change the existing wording to) that improve clarity in different sections of the manuscript (see the foot of this letter). Please attend to these test modifications from the Academic Editor.

b) Please address my Data Policy requests below; specifically, we need you to supply the numerical values underlying Figs 1ABCD, 2ABCDEF, 3BCDE, 4CEFHIKLNOP, 5ABC, S1ABC, S2BC, S3BCDEF, S5CDEFGHIJL, S6ABCDEFG either as a supplementary data file or as a permanent DOI’d deposition (such as Zenodo).

c) Please clearly cite the location of the data in all relevant main and supplementary figure legends, for example: “The data underlying this figure can be found at doi.org/10.xxx/zenodo.xxxxx.” We understand that this may appear repetitive, but it helps make the figures and their legends more self contained.

d) Please include the URLs of your funders in the Financial Disclosure statement.

e) Please make any custom code available, either as a supplementary file or as part of your data deposition.

f). "Significance Statement" - we don't do these at PLOS Biology. So, please delete it.

**Suggested modifications by AE (Lightly Edited)**

Title: Change the title to:

siRNA-guided DNA methylation at a sucrose transporter gene potentially contributes to climatic adaptation in plants

Abstract:

Replace “Exploration of methylome data and epigenome editing experiments indicated that epigenetic marks on SUC2’s exon 2 could enact the climate-dependent expression of SUC2.” with “Exploration of methylome data and epigenome editing experiments suggested that epigenetic marks on SUC2’s exon 2 contributed to the climate-dependent expression of SUC2.”

Replace “Besides illustrating a novel regulatory strategy of carbon export from leaves, our results establish exon methylation mediated by gene-derived siRNAs as a mechanism for plant adaptation.” with “Besides illustrating a novel regulatory strategy of carbon export from leaves, our results establish exon methylation mediated by gene-derived siRNAs as yet another potential mechanism for plant adaptation.”

Results:

Line227-228:

Instead of “Interestingly …” write “met1 mutants lack all CG methylation, which was accompanied by fewer CHH reads in source leaves and less CHH methylation on exon 2 of SUC2, although there was not a robust effect on SUC1 expression (p=0.098).”

As you address these items, please take this last chance to review your reference list to ensure that it is complete and correct. If you have cited papers that have been retracted, please include the rationale for doing so in the manuscript text, or remove these references and replace them with relevant current references. Any changes to the reference list should be mentioned in the cover letter that accompanies your revised manuscript.

In addition to these revisions, you may need to complete some formatting changes, which you will receive in a follow up email. A member of our team will be in touch with a set of requests shortly. If you do not receive a separate email within a few days, please assume that checks have been completed, and no additional changes are required.

We expect to receive your revised manuscript within two weeks.

To submit your revision, please go to https://www.editorialmanager.com/pbiology/ and log in as an Author. Click the link labelled 'Submissions Needing Revision' to find your submission record. Your revised submission must include the following:

- a cover letter that should detail your responses to any editorial requests, if applicable, and whether changes have been made to the reference list

- a Response to Reviewers file that provides a detailed response to the reviewers' comments (if applicable, if not applicable please do not delete your existing 'Response to Reviewers' file.)

- a track-changes file indicating any changes that you have made to the manuscript.

NOTE: If Supporting Information files are included with your article, note that these are not copyedited and will be published as they are submitted. Please ensure that these files are legible and of high quality (at least 300 dpi) in an easily accessible file format. For this reason, please be aware that any references listed in an SI file will not be indexed. For more information, see our Supporting Information guidelines:

https://journals.plos.org/plosbiology/s/supporting-information

*Published Peer Review History*

Please note that you may have the opportunity to make the peer review history publicly available. The record will include editor decision letters (with reviews) and your responses to reviewer comments. If eligible, we will contact you to opt in or out. Please see here for more details:

https://plos.org/published-peer-review-history/

*Press*

Should you, your institution's press office or the journal office choose to press release your paper, please ensure you have opted out of Early Article Posting on the submission form. We ask that you notify us as soon as possible if you or your institution is planning to press release the article.

*Protocols deposition*

To enhance the reproducibility of your results, we recommend that if applicable you deposit your laboratory protocols in protocols.io, where a protocol can be assigned its own identifier (DOI) such that it can be cited independently in the future. Additionally, PLOS ONE offers an option for publishing peer-reviewed Lab Protocol articles, which describe protocols hosted on protocols.io. Read more information on sharing protocols at https://plos.org/protocols?utm_medium=editorial-email&utm_source=authorletters&utm_campaign=protocols

Please do not hesitate to contact me should you have any questions.

Sincerely,

Ankiit

Ankiit Ahluwalia,

aahluwalia@plos.org

PLOS Biology

------------------------------------------------------------------------

****[DELETE AS APPROPRIATE]****

ETHICS STATEMENT:

-- Please include the full name of the IACUC/ethics committee that reviewed and approved the animal care and use protocol/permit/project license. Please also include an approval number.

-- Please include the specific national or international regulations/guidelines to which your animal care and use protocol adhered. Please note that institutional or accreditation organization guidelines (such as AAALAC) do not meet this requirement.

-- Please include information about the form of consent (written/oral) given for research involving human participants. All research involving human participants must have been approved by the authors' Institutional Review Board (IRB) or an equivalent committee, and must have been conducted according to the principles expressed in the Declaration of Helsinki.

------------------------------------------------------------------------

DATA POLICY:

You may be aware of the PLOS Data Policy, which requires that all data be made available without restriction: http://journals.plos.org/plosbiology/s/data-availability. For more information, please also see this editorial: http://dx.doi.org/10.1371/journal.pbio.1001797

Note that we do not require all raw data. Rather, we ask that all individual quantitative observations that underlie the data summarized in the figures and results of your paper be made available in one of the following forms:

1) Supplementary files (e.g., excel). Please ensure that all data files are uploaded as 'Supporting Information' and are invariably referred to (in the manuscript, figure legends, and the Description field when uploading your files) using the following format verbatim: S1 Data, S2 Data, etc. Multiple panels of a single or even several figures can be included as multiple sheets in one excel file that is saved using exactly the following convention: S1_Data.xlsx (using an underscore).

2) Deposition in a publicly available repository. Please also provide the accession code or a reviewer link so that we may view your data before publication.

Regardless of the method selected, please ensure that you provide the individual numerical values that underlie the summary data displayed in the following figure panels as they are essential for readers to assess your analysis and to reproduce it:

[Figs….]

NOTE: the numerical data provided should include all replicates AND the way in which the plotted mean and errors were derived (it should not present only the mean/average values).

Please also ensure that figure legends in your manuscript include information on where the underlying data can be found, and ensure your supplemental data file/s has a legend.

Please ensure that your Data Statement in the submission system accurately describes where your data can be found.

------------------------------------------------------------------------

APPROPRIATE DATA PRESENTATION?

(If you think the paper is not using best practice, add the following)

- Please ensure that you are using best practice for statistical reporting and data presentation. These are our guidelines https://journals.plos.org/plosbiology/s/best-practices-in-research-reporting#loc-statistical-reporting and a useful resource on data presentation https://journals.plos.org/plosbiology/article?id=10.1371/journal.pbio.1002128

- If you are reporting experiments where n ≤ 5, please plot each individual data point.

------------------------------------------------------------------------

CODE POLICY

Per journal policy, if you have generated any custom code during the course of this investigation, please make it available without restrictions. Please ensure that the code is sufficiently well documented and reusable, and that your Data Statement in the Editorial Manager submission system accurately describes where your code can be found. More information on our Code Policy, what and how to share can be found here: https://journals.plos.org/plosbiology/s/code-availability

Please note that we cannot accept sole deposition of code in GitHub, as this could be changed after publication. However, you can archive this version of your publicly available GitHub code to Zenodo. Once you do this, it will generate a DOI number, which you will need to provide in the Data Accessibility Statement (you are welcome to also provide the GitHub access information). See the process for doing this here: https://docs.github.com/en/repositories/archiving-a-github-repository/referencing-and-citing-content

------------------------------------------------------------------------

SPECIES INDICATED IN THE ABSTRACT?

- Please note that per journal policy, the model system/species studied should be clearly stated in the abstract of your manuscript.

**EDITOR, CONSIDER ALSO INCLUDING ORGANISM IN THE TITLE, IF SENSICAL/RELEVANT AND/OR TO AVOID BEING MISLEADING/OVERREACHING (not always preferred)**

------------------------------------------------------------------------

BLOT AND GEL REPORTING REQUIREMENTS:

We require the original, uncropped and minimally adjusted images supporting all blot and gel results reported in an article's figures or Supporting Information files. We will require these files before a manuscript can be accepted so please prepare and upload them now. Please carefully read our guidelines for how to prepare and upload this data: https://journals.plos.org/plosbiology/s/figures#loc-blot-and-gel-reporting-requirements

------------------------------------------------------------------------

DATA NOT SHOWN?

- Please note that per journal policy, we do not allow the mention of "data not shown", "personal communication", "manuscript in preparation" or other references to data that is not publicly available or contained within this manuscript. Please either remove mention of these data or provide figures presenting the results and the data underlying the figure(s).

------------------------------------------------------------------------

Reviewer remarks:

# Author comment: Author response to Decision Letter 3

**DOI:** 10.1371/journal.pbio.3003919.r005

2 Jul 2026

Attachment Submitted filename: Response_3.docx

# Editor report: Decision Letter 3

**DOI:** 10.1371/journal.pbio.3003919.r006

**Authors:** Ankiit Ahluwalia

10 Jul 2026

Dear Dr Liesche,

Thank you for the submission of your revised Research Article "siRNA-guided DNA-methylation at a sucrose transporter gene potentially contributes to climatic plant adaptation in plants" for publication in PLOS Biology. On behalf of my colleagues and the Academic Editor, Detlef Weigel, I am pleased to say that we can in principle accept your manuscript for publication, provided you address any remaining formatting and reporting issues. These will be detailed in an email you should receive within 2-3 business days from our colleagues in the journal operations team; no action is required from you until then. Please note that we will not be able to formally accept your manuscript and schedule it for publication until you have completed any requested changes.

Please take a minute to log into Editorial Manager at http://www.editorialmanager.com/pbiology/, click the "Update My Information" link at the top of the page, and update your user information to ensure an efficient production process.

PRESS

We frequently collaborate with press offices. If your institution or institutions have a press office, please notify them about your upcoming paper at this point, to enable them to help maximise its impact. If the press office is planning to promote your findings, we would be grateful if they could coordinate with biologypress@plos.org. If you have previously opted in to the early version process, we ask that you notify us immediately of any press plans so that we may opt out on your behalf.

We also ask that you take this opportunity to read our Embargo Policy regarding the discussion, promotion and media coverage of work that is yet to be published by PLOS. As your manuscript is not yet published, it is bound by the conditions of our Embargo Policy. Please be aware that this policy is in place both to ensure that any press coverage of your article is fully substantiated and to provide a direct link between such coverage and the published work. For full details of our Embargo Policy, please visit http://www.plos.org/about/media-inquiries/embargo-policy/.

Thank you again for choosing PLOS Biology for publication and supporting Open Access publishing. We look forward to publishing your study.

Sincerely,

Ankiit Ahluwalia,

PLOS Biology

aahluwalia@plos.org
